# Association between high serum blood glucose lymphocyte ratio and all-cause mortality in non-traumatic cerebral hemorrhage: a retrospective analysis of the MIMIC-IV database

**DOI:** 10.3389/fendo.2023.1290176

**Published:** 2023-11-29

**Authors:** Shiqiang Yang, Yanwei Liu, Shiqiang Wang, Zhonghai Cai, Anqiang Yang, Xuhui Hui

**Affiliations:** ^1^ Department of Neurosurgery, First People’s Hospital of Yibin, Yibin, Sichuan, China; ^2^ Department of Neurosurgery, West China Hospital, Sichuan University, Chengdu, Sichuan, China; ^3^ Department of Neurology, First People’s Hospital of Yibin, Yibin, Sichuan, China; ^4^ Department of Neuro-Oncology, Cancer Hospital, Chongqing University, Chongqing, China

**Keywords:** glucose-to-lymphocyte ratio, nontraumatic cerebral hemorrhage, medical information mart for intensive care IV, mortality, linear relationship, intensive care unit

## Abstract

**Background:**

This study aimed to evaluate the association between the glucose-to-lymphocyte ratio (GLR) and all-cause mortality in intensive care unit (ICU) patients with Non-traumatic cerebral hemorrhage.

**Methods:**

This is a retrospective cohort study. Baseline data and in-hospital prognosis from patients with non-traumatic cerebral hemorrhage admitted to the intensive care unit. Multivariate COX regression analysis was applied and adjusted hazard ratios (HR) and 95% predictive values with confidence intervals (CI) were calculated. Survival curves for the two groups of cases were plotted using K-M curves, and subgroup analyses were performed in one step. Using restricted cubic spline curves, we analyzed the potential linear relationship between GLR and outcome indicators.

**Results:**

In the Medical Information Mart for Intensive Care IV (MIMIC-IV database), we extracted 3,783 patients with nontraumatic intracerebral hemorrhage, and 1,806 patients were finally enrolled in the study after exclusion of missing values and patients with a short hospital stay. The overall ICU mortality rate was 8.2% (148/1806) and the in-hospital mortality rate was 12.5% (225/1806). The use of curve fitting yielded a significant linear relationship between GLR and both ICU mortality and in-hospital mortality. It also suggested a reference point at GLR=3.9. These patients were categorized into high and low subgroups based on the median value of their GLR (GLR = 3.9). Model comparisons based on multivariate COX regression analysis showed that in-hospital mortality was higher in the high GLR group after adjusting for all confounders (HR = 1.31, 95% CI: 1.04-1.47), while the ICU mortality in the high GLR group was (HR = 1.73, 95% CI: 1.18-2.52). Stratified analyses based on age, gender, race, GCS, BMI, and disease type showed stable correlations between the high GLR group and in-hospital and ICU mortality.

**Conclusion:**

Based on our retrospective analysis, it is known that as the GLR increased, the in-hospital mortality rate and ICU mortality rate of patients with nontraumatic cerebral hemorrhage also increased progressively in the United States in a clear linear relationship. However, further studies are needed to confirm these findings.

## Introduction

Acute non-traumatic cerebral hemorrhage, including diseases such as hypertensive cerebral hemorrhage, spontaneous subarachnoid hemorrhage, and hemorrhage of auto-vascular causes, is a group of diseases that seriously endanger the lives of patients. It ranks as the second most prevalent type of stroke, severity, swift advancement, elevated rates of mortality and disability, thereby constituting a significant peril to the global population (1, 2). Despite the implementation of optimal care within the intensive care unit and during hospitalization, patients afflicted with nontraumatic intracerebral hemorrhage continue to exhibit a considerable in-hospital mortality rate (3).Epidemiological surveys have shown that the in-hospital mortality rate for non-traumatic intracerebral hemorrhage is as high as 20%, and even higher in developing countries (3, 4). Considering the serious life-threatening nature of this group of patients, there is an urgent need for non-invasive and inexpensive tests to identify those at greater risk of death and to prevent death (5).

Numerous clinical studies have determined that patients who experience intracerebral hemorrhage exhibit a concurrent systemic inflammatory response. Furthermore, patients with severe cerebral hemorrhage have demonstrated signs of immune cell activation and abnormal host reactions (6, 7). Moreover, various systemic inflammatory biomarkers, such as the neutrophil-lymphocyte ratio (NLR) (8), platelet-lymphocyte ratio (PLR) (9), and lymphocyte-monocyte ratio (LMR) (10), have been linked to critical cerebrovascular disease and unfavorable prognosis in patients. The presence of immune cell deficiency and dysfunction is widely recognized as significant contributors to secondary infections and unfavorable prognosis in critically ill patients. Consequently, variations in the quantity and functionality of immune cells may be linked to mortality rates in this patient population. Among the key effector cells implicated in the systemic inflammatory response of critically ill patients, lymphocytes play a prominent role (11). Consequently, lymphocyte counts, serving as indicators of immune system status, appear to hold predictive value for the prognosis of critically ill patients suffering from intracerebral hemorrhage (12).

Furthermore, Patients with acute cerebral hemorrhage are at increased risk of stress hyperglycemia of varying intensity, and glycemic management may be challenging. A maladaptive mechanism caused by acute stress and inflammatory states antagonizes insulin-mediated glucose uptake through excess cortisol. In addition to hormonal changes, studies have found that cytokines such as TNF-alpha and interleukin-1 are involved in the dysregulation of insulin signaling. Patients progressively develop a hyperglycemic state after the onset of the disease. In addition, it has been shown that there is a correlation between hyperglycemia and poor prognosis in intensive care unit patients, including increased mortality, hospital-acquired infections, wound complications, prolonged intensive care unit stays, and an increased incidence of intensive care neuropathy (11, 13).Acute hyperglycemia, in particular, emerges as an autonomous risk factor for mortality in critically ill individuals (14). Differences between the two indicators, blood glucose and absolute blood lymphocyte values, become apparent through changes in the GLR, an increase in the GLR implying an imbalance in glucose regulation and immune response. This disparity results in the occurrence of organ failure, metabolic disturbances, compromised immune function, and an imbalance between oxygen availability and demand, ultimately culminating in mortality. Increasing evidence suggests a significant correlation between heightened blood glucose levels and diminished lymphocyte counts, indicating the severity of critical cerebral hemorrhage in patients (15). The elevated GLR may serve as an indicator of the combined impact of hyperglycemia and immune dysfunction in critically ill individuals. Current clinical studies suggest that elevated GLR is an important predictor of acute mortality and prognosis in patients with gastric cancer (16),hepatocellular carcinoma (17), breast cancer, thyroid cancer, rectal cancer (18), acute respiratory distress syndrome and acute exacerbation of chronic obstructive pulmonary disease (11).Therefore, it has important clinical significance in terms of malignant tumor disease burden and respiratory disease burden. The aim of this study was to evaluate the correlation between GLR at admission and prognosis of hospitalization in patients with non-traumatic cerebral hemorrhage. GLR, a composite measure encompassing both glucose levels and systemic inflammation, may offer a novel basis and benchmark for the clinical treatment of individuals with severe cerebral hemorrhage. This will contribute to the early identification of critically ill patients in the clinic by these easily available biomarkers and individualized targeted therapy to save patients’ lives to a greater extent.

## Materials

### Study population

**Figure 1:**
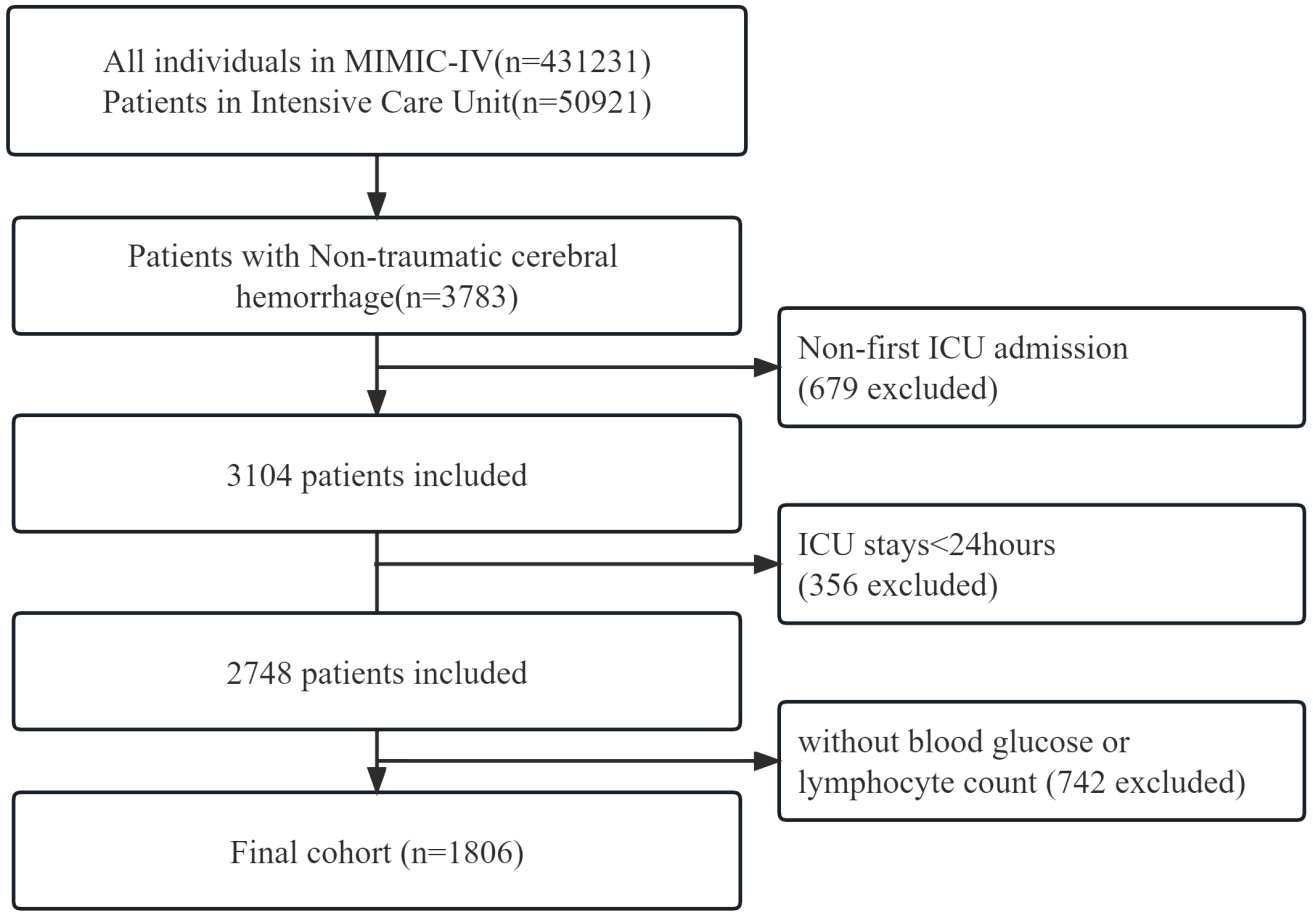
A flowchart of study patients.

This retrospective cohort study adhered to the Guidelines for Enhancing the Reporting of Observational Studies in Epidemiology. The researchers accessed health-related data from the MIMIC-IV (version 2.2) database, a comprehensive and well-maintained general-purpose database created by the MIT Computational Physiology Laboratory. This database contains comprehensive and reliable medical records of patients admitted to the Intensive Care Unit at Beth Israel Deaconess Medical Center. One author (Shiqiang Yang) complied with requirements for access to the database and was responsible for the data extraction(certification number 52945707). Patients diagnosed with non-traumatic cerebral hemorrhage according to the International Classification of Diseases, 9th and 10th editions, were included in this study. Between 2008 and 2019, over 50,000 adult patients were admitted to the ICU at Beth Israel Deaconess Medical Center, Boston. Of these, a total of 3783 patients with non-traumatic cerebral hemorrhage were selected based on records from ICD-9 codes 430 and 431, and I60, I601 ~ I609 and I610 ~ I619 in ICD10 codes. Exclusion criteria were as follows: First, we excluded 679 patients who were not admitted to the ICU for the first time from all 3783 data. Second, 356 patients who were admitted to the ICU for less than 24 hours were excluded from the remaining data. Finally, 742 patients without clear blood glucose and blood lymphocyte counts were excluded. We provided adequate explanations for the exclusion of patients. Finally, a total of 1,806 patients were included in this study (**Figure 1**).

### Data collection

The data for this study was collected by executing Structured Query Language (SQL) using PostgresSQL (version 13.9.9) and Navicate Premium (version 16.1.7) software. The variables selected for analysis can be classified into five main categories: (1) demographic characteristics such as age, gender, weight, height, and BMI; (2) vital signs encompassing systolic blood pressure, diastolic blood pressure, respiration rate, heart rate, temperature, oxygen saturation, length of stay in the intensive care unit (ICU) and hospital, and ICU and hospital mortality.(3) Various scoring systems such as the Glasgow Score (GCS), Sequential Organ Failure Score (SOFA), and Logistic Organ Dysfunction System (LODS) are utilized. (4) Comorbidities encompass pulmonary disease, coagulation abnormalities, heart failure, renal disease, and liver disease. (5) Laboratory indicators encompass blood glucose, white blood cells (WBC), hemoglobin, platelets, serum sodium, serum creatinine, PTT, anion gap, bicarbonate, chloride, and other relevant factors.

The follow-up period began upon admission and ended upon death for deceased patients or discharge for surviving patients. The GLR index was calculated by dividing the fasting blood glucose (mmol/L) by the serum lymphocyte (10^9 cells/L) on the first day of admission. All laboratory variables and disease severity scores were obtained from data recorded for the first instance after the patient’s admission to the intensive care unit. To minimize potential bias, when missing values were found for the glucose, serum lymphocyte, and death outcome variables during cleaning of the raw data, we deleted this one case. Covariates with missing values exceeding 10% were excluded. Covariates with less than 10% missing data were processed by the multiple interpolation scheme of the Free Statistics software version 1.7 (Beijing, China) and the statistical software packages R 3.3.2. (**Table 1**).

**Table 1:** Details of missing values.

Variables	The number of missing values	The percent of missing values(%)
Hemoglobin	17	0.94%
Platelets	21	1.16%
Sodium	25	1.38%
Potassium	26	1.43%
Bicarbonate	27	1.49%
Aniongap	34	1.88%
Chloride	35	1.94%
Calcium	41	2.21%
partial thromboplastin time	64	3.54%
Blood glucose	92	5.09%
Blood lymphocyte	650	35.99%

### Clinical outcomes

The primary endpoint of this study was all-cause mortality in the ICU, and the second endpoint was all-cause mortality in hospital.

### Statistical analysis

Continuous variables were reported as mean ± standard deviation (SD) or median interquartile range (IQR), while categorical variables were expressed as percentages. The patients were divided into two groups based on the GLR index using the upper quartile. The Q1 group included patients with a low index (GLR<3.9, n=843), while the Q2 group consisted of patients with a high index (BAR≥3.9, n=963). Fisher’s exact test, chi-square test, or Kruskal-Wallis test were used to assess the statistical differences between the two groups for each variable. The study employed the restricted cubic splines regression analysis to elucidate the linear correlation between the GLR index and both ICU and hospital all-cause mortality in individuals suffering from acute nontraumatic cerebral hemorrhage.

Additionally, a multivariate Cox proportional hazards model was utilized to evaluate the relationship between the GLR index and both ICU mortality and in-Hospital mortality. Confounding variables were chosen through a combination of clinical expertise, previous research, and effect values surpassing 10% for baseline variables. In the crude Model, the covariates were left unadjusted. In Model I, the covariates were adjusted for age, sex, race, and BMI. In Model II, the covariates were adjusted for the covariates in Model I, along with Glasgow Coma Scale score, Sequential Organ Failure Assessment score, and Logistic Organ Dysfunction System score. In Model III, the covariates from Model II were adjusted for, along with additional variables including WBC, aniongap, bicarbonate, BUN, chloride, creatinine, sodium, PTT, hemoglobin, and platelets. A sensitivity analysis was conducted to assess the reliability of the data analysis.

Kaplan-Meier (K-M) survival curves were utilized to visually depict the relationship between two GLR groups and the occurrence of ICU mortality and in-hospital mortality. Moreover, the study conducted interaction and stratification analyses, considering variables such as age, sex, race, BMI, GCS, and type of disease. The results were presented as hazard ratios (HR) accompanied by a 95% confidence interval (CI), and statistical significance was determined by p values less than 0.05. The statistical software packages R 3.3.2 and Free Statistics software version 1.7.1 (Beijing, China) were employed for all statistical analyses.

## Results

### Population

In total, 3783 patients were identified according to the non-traumatic cerebral hemorrhage criterion. Of these, 1977 patients without GLR values and other specific conditions were excluded, and 1806 with non-traumatic cerebral hemorrhage criterion were included in the final cohort (**Figure 1** shows a flow chart).Out of 1806 patients, 148 patients died in the ICU with a rate of 8.2. In total, 225 patients had a fatal outcome during hospitalization with an incidence rate of 12.5%.

### Baseline characteristics

This study included a cohort of 1806 patients out of the total 3783 individuals diagnosed with non-traumatic cerebral hemorrhage and undergoing treatment in the intensive care unit (ICU), as depicted in **Figure 1**. Among these patients, 1043 were males, with an average age of 64.7 ± 16.9 years. The patients were divided into two groups based on their GLR index, and the distribution of baseline population characteristics for all patients and subgroups can be found in **Table 1**. Patients in the high GLR index group (≥3.9) exhibited significantly higher scores in SOFA and LODS, as well as higher rates of ICU and in-hospital mortality compared to the low GLR index group. The detailed Baseline characteristics of this study is detailed in **Table 2**.

**Table 2:** The clinical characteristics of patients with non-traumatic intracranial hemorrhage.

Characteristics	GLR: blood glucose-to-lymphocyte ratio			*p-*value
	Total (n = 1806)	Tertile1 (<3.9) (n = 843)	Tertile2 ((≥3.9) (n = 963)	
Age, years	64.7 ± 16.9	63.8 ± 17.5	65.5 ± 16.5	0.038
Gender, Male (%)	1043 (57.8)	497 (59)	546 (56.7)	0.332
Race, White (%)	1200 (66.4)	588 (69.8)	612 (63.6)	0.005
BMI,Kg/m^2^	27.5 ± 5.4	27.2 ± 4.8	27.8 ± 5.9	0.025
SOAI, hours	4.7 (3.0, 6.0)	4.8 (2.0, 4.0)	4.4 (2.0, 5.5)	0.151
Vital signs				
SBP,mmHg	118.4 ± 17.2	117.9 ± 16.7	118.9 ± 17.7	0.234
DBP,mmHg	64.0 ± 11.6	63.6 ± 11.5	64.3 ± 11.7	0.239
MBP,mmHg	78.6 ± 11.6	78.1 ± 11.4	79.0 ± 11.8	0.131
RR,beats/min	19.5 ± 3.9	19.1 ± 3.6	19.8 ± 4.1	< 0.001
HR,beats/min	85.6 ± 15.6	84.1 ± 15.0	86.9 ± 15.9	< 0.001
Temperature,°C	37.1 ± 0.7	37.0 ± 0.7	37.1 ± 0.8	0.008
SpO2, (%)	96.8 ± 2.4	97.0 ± 2.2	96.7 ± 2.5	0.028
Hospitalization status				
ICU length of stay, days	2.0 (1.1, 4.0)	2.0 (1.1, 3.8)	2.1 (1.1, 4.1)	0.127
ICU mortality, n (%)	148 (8.2)	40 (4.7)	108 (11.2)	< 0.001
Hospital length of stay, days	7.1 (4.0, 12.1)	7.0 (4.1, 12.1)	7.4 (4.0, 12.2)	0.609
Hospital mortality, n (%)	225 (12.5)	84 (10)	141 (14.6)	0.003
Scoring systems				
GCS	12.7 ± 3.4	12.8 ± 3.3	12.7 ± 3.4	0.539
SOFA	5.3 ± 3.8	5.0 ± 3.6	5.6 ± 3.9	< 0.001
LODS	4.8 ± 3.3	4.4 ± 3.1	5.1 ± 3.4	< 0.001
Comorbidities, n (%)				
Chronic pulmonary disease, n (%)	319(17.7)	132(15.7)	187(19.4)	0.031
Coagulation abnormality, n (%)	301(16.7)	133(15.8)	168(17.4)	0.52
Liver diseases, n (%)	185(10.2)	80(9.5)	105(10.9)	0.141
Cardiovascular diseases, n (%)	398(22.0)	170(20.2)	228(23.7)	0.006
Malignancy, n (%)	124 (6.9)	47 (5.6)	77 (8)	0.304
Renal diseases, n (%)	317(17.6)	136(16.1)	181(18.8)	0.008
Laboratory tests				
Hemoglobin, (g/dL)	11.4 ± 2.2	11.4 ± 2.2	11.5 ± 2.2	0.624
Platelets, (10^9^/L)	235.1 ± 68.1	234.8 ± 60.5	235.3 ± 64.5	0.936
WBC, (10^9^/L)	14.1 ± 10.0	14.4 ± 11.4	13.8 ± 8.5	0.252
Anion gap	17.0 ± 5.2	16.0 ± 4.4	17.8 ± 5.6	< 0.001
Bicarbonate, (mmol/L)	24.6 ± 4.6	24.7 ± 4.5	24.4 ± 4.7	0.216
Creatinine, (mmol/L)	1.7 ± 2.0	1.6 ± 2.0	1.7 ± 1.9	0.297
Sodium, (mmol/L)	139.8 ± 5.1	139.5 ± 4.7	140.1 ± 5.4	0.02
PTT, seconds	44.0 ± 31.4	42.4 ± 29.1	45.4 ± 33.2	0.042
Glucose,(mmol/L)	7.9 (6.4, 10.6)	6.8 (5.8, 8.2)	9.4 (7.2, 13.2)	< 0.001
Lymphocytes,(10^9^/L)	2.1 (1.4, 3.0)	2.9 (2.3, 3.5)	1.4 (1.1, 2.0)	< 0.001

GLR:serum blood glucose/lymphocyte count; BMI,Body mass index; SOAI, stroke onset to the admission to ICU; SBP, systolic blood pressure; DBP, diastolic blood pressure; MBP, mean blood pressure; RR, respiratory rate; HR, heart rate; SpO2, percutaneous oxygen saturation; GCS, Glasgow Coma Score; SOFA, Sequential organ function score; LODS, The Logistic organ dysfunction system; WBC, white blood cell; PTT, Partial thromboplastin time.

### Curve fitting analysis

**Figure 2:**
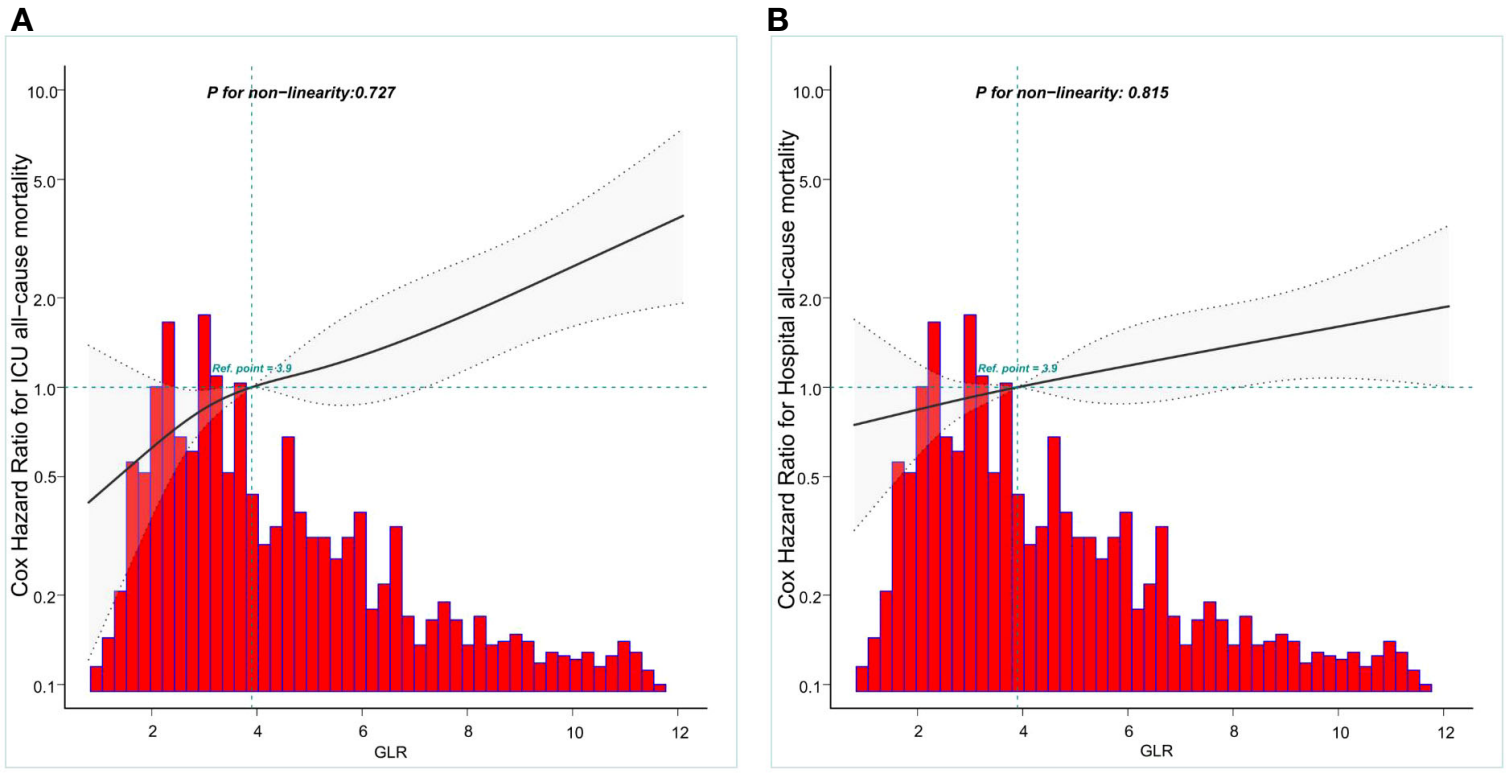
Construction of smooth curve describing the risk of mortality against GLR using a restricted cubic spline model. **(A)** ICU all-cause mortality; **(B)** Hospital all-cause mortality. The solid black line represents the smooth curve fit between variables. Grey bands present the 95% confidence interval. Data were adjusted for age, gender, race, BMI,GCS, SOFA,LODS,hemoglobin, Platelets, WBC,anion gap,bicarbonate,BUN, chloride, creatinine sodium and partial thromboplastin time.

Restricted cubic spline models were utilized to construct smooth curves representing the mortality risk indexed by GLR for both ICU all-cause mortality and hospital all-cause mortality. The solid black line depicts the smooth curve fit between the variables, while the gray bands indicate the 95% confidence intervals. Following the adjustment for covariates, a statistically significant association was observed between GLR levels and all-cause mortality in both the ICU and hospital settings. Specifically, the all-cause mortality in patients with nontraumatic cerebral hemorrhage exhibited a linear increase with higher GLR levels, as depicted in **Figure 2**. The two curves were separately analyzed to identify inflection points.

### Kaplan–Meier curves

**Figure 3:**
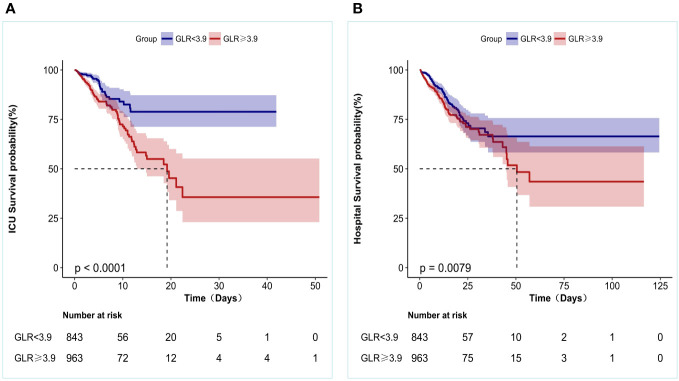
Kaplan-Meier survival curves for patients with Non-traumatic intracranial hemorrhage based on GLR group. **(A)** ICU all-cause mortality; **(B)** Hospital all-cause mortality. x-Axis: survival time (days). y-Axis: survival probability.

In addition, KM survival curves showed that patients in the high GLR score group (GLR≥3.9) at admission had lower ICU survival and in-Hospital survival (both p < 0.05), as shown in **Figure 3**.

### Univariate Cox regression analysis

In this study, a univariate Cox regression analysis was conducted to examine the independent effects of various variables on ICU mortality and in-hospital mortality. The findings indicated statistically significant effects of age, GCS, LODS, SOFA, blood glucose, SBP, DBP, MBP, Temperature, respiratory rate, heart rate, SpO2, WBC, Anion gap, Bicarbonate, and BUN, along with GLR (all p<0.05, **Table Supplementary 1**).

### Multi-variable Cox regression analysis

In this study, three multivariate Cox regression models were constructed to examine the independent impact of GLR on in-ICU and Hospital mortality. The resulting effect sizes (HRs) and their corresponding 95% confidence intervals are presented in **Table 3**. It was observed that the unadjusted model HRs remained statistically significant (p < 0.05) across all three models. Specifically, in the unadjusted model, a one-unit increase in GLR was found to be associated with a 5% increase in the difference in ICU mortality (HR = 1.05, 95% CI: 1.02-1.08).In the minimally adjusted model (model I), an increase of one unit in GLR was found to be associated with a 5% increase in the difference in ICU mortality (HR = 1.05, 95% CI: 1.02-1.08). In Model II, which was further adjusted for Model I + GCS + SOFA + LODS, the difference in ICU mortality increased by 3% for each unit increase in GLR (HR = 1.03, 95% CI: 1-1.06).However, in the fully adjusted model (Model III), which accounted for various covariates such as age, gender, race, GCS, SOFA, LODS, pneumonia, stroke onset to the admission to ICU,hemoglobin, platelets, WBC, anion gap, bicarbonate, BUN, chloride, creatinine, sodium, and PTT, the difference in in-ICU mortality increased by 2% for each unit increase in GLR (HR = 1.02 CI: 0.97 to 1.04).In the unadjusted model, the effect value of GLR on hospitalization mortality was (HR = 1.02, 95% CI: 1.01-1.05). In the minimally adjusted model (Model I), the effect value was (HR = 1.02, 95% CI: 1-1.05). In model II,the effect value was (HR = 1.01, 95% CI: 0.98-1.04).In the fully adjusted model (Model III), the effect size was (HR =0.99, 95% CI: 0.96-1.02).

**Table 3:** Multivariable cox regression models evaluating the association between GLR and ICU and Hospital all-cause mortality.

Variable	Crude		Model I		Model II		Model III	
	HR (95%CI)	*P*-value	HR (95%CI)	*P*-value	HR (95%CI)	*P*-value	HR (95%CI)	*P*-value
ICU all-cause mortality								
GLR	1.05 (1.02~1.08)	0.001	1.05 (1.02~1.08)	0.001	1.03 (1~1.06)	0.063	1.02 (0.97-1.04)	0.673
GLR<3.9	1 (Ref)		1 (Ref)		1 (Ref)		1 (Ref)	
GLR≥3.9	2.25 (1.56~3.23)	<0.001	2.19 (1.52~3.15)	<0.001	1.85 (1.28~2.67)	0.001	1.71 (1.16~2.54)	0.005
Hospital all-cause mortality								
GLR	1.02 (1.01~1.05)	0.002	1.02 (1~1.05)	0.099	1.01 (0.98~1.04)	0.56	0.99 (0.97~1.03)	0.531
GLR<3.9	1 (Ref)		1 (Ref)		1 (Ref)		1 (Ref)	
GLR≥3.9	1.44 (1.19~1.89)	0.008	1.4 (1.17~1.83)	0.015	1.42 (1.13~1.61)	0.018	1.33 (1.04~1.49)	0.032

>Crude model: adjusted for none; Model I: adjusted for age, gender, race and BMI; Model II:adjusted for Model I +GCS+SOFA+LODS;

Model III: adjusted for Model II +SOAI+hemoglobin+Platelets+WBC+anion gap+bicarbonate+BUN+chloride+creatinine+sodium+PTT.

To conduct further sensitivity analyses, we transformed the continuous variable GLR into a categorical variable (median GLR), with the low GLR group (Q1) serving as the baseline reference. The association between the categorical variable GLR and intensive care unit and hospitalized all-cause mortality was evaluated using the low subgroup (GLR <3.9) as the reference group. In the initial analysis, the high subgroup (GLR ≥3.9) exhibited a significantly elevated risk of intensive care unit (HR: 2.25, 95% CI: 1.56 to 3.23) and in-hospital all-cause mortality (HR: 1.44, 95% CI: 1.19 to 1.89).In the minimally adjusted model I, the heightened risk of ICU mortality (HR: 2.19, 95% CI: 1.52 to 3.15) and in-hospital all-cause mortality (HR: 1.4, 95% CI: 1.17 to 1.83) remained statistically significant even after controlling for age, sex, race, and body mass index. In Model II, which was further adjusted for Model I + GCS + SOFA + LODS, the hazard ratios for ICU mortality (HR: 1.85, 95% CI: 1.28 to 2.67) and hospitalized all-cause mortality (HR: 1.42, 95% CI: 1.13 to 1.61) continued to demonstrate significance. Similarly, in the fully adjusted Model III, the hazard ratios for ICU mortality (HR: 1.71, 95% CI: 1.16 to 2.54) and hospitalized all-cause mortality (HR: 1.33, 95% CI: 1.04 to 1.49) continued to demonstrate significance. Thus, the heightened risk of mortality in patients belonging to the high GLR group remained statistically significant.

### Subgroup analysis

**Figure 4:**
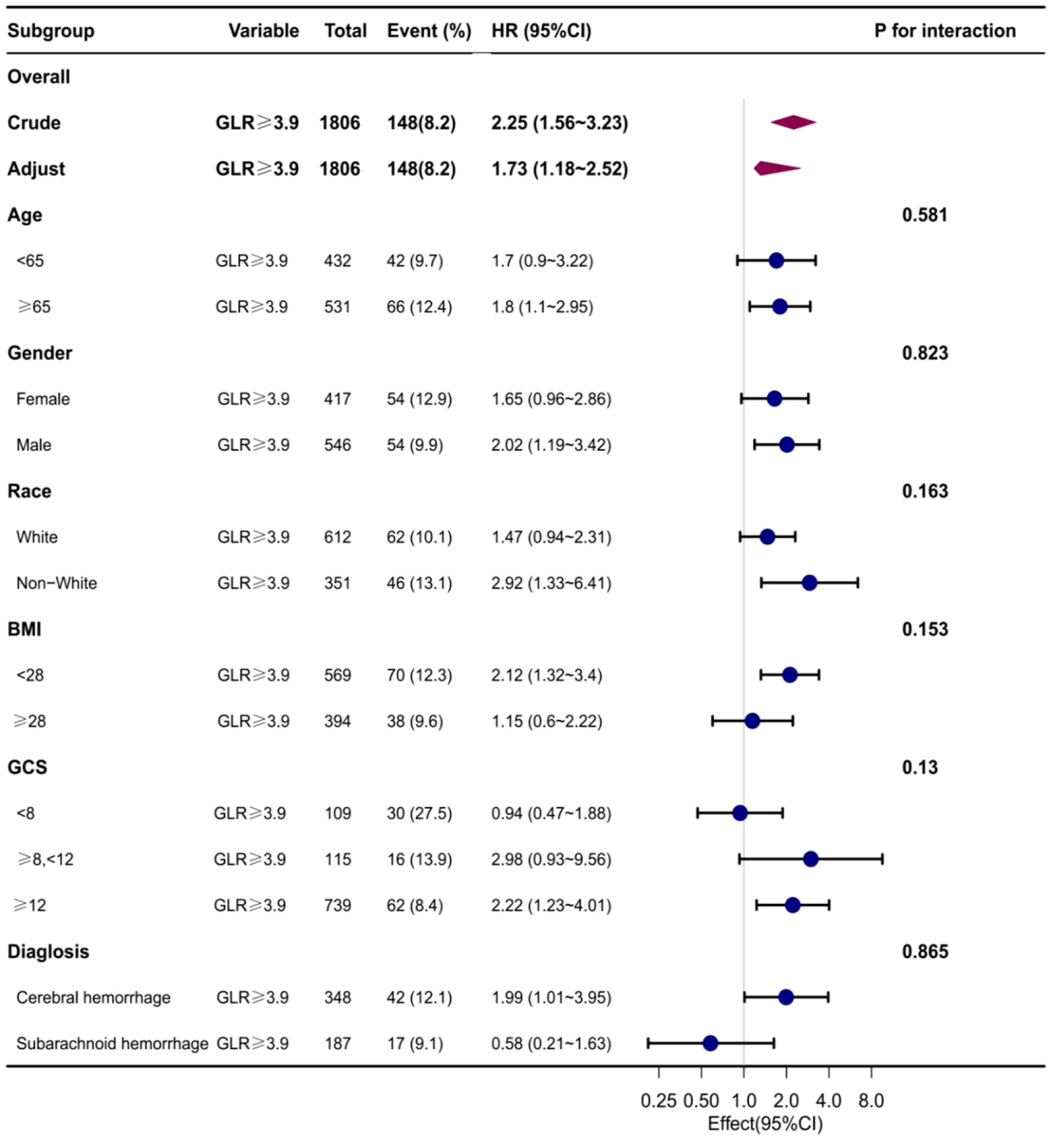
Subgroup analyses of the effect of on ICUall-cause mortality. Data were adjusted for age, gender, race, BMI, GCS, SOFA, LODS, hemoglobin, Platelets, WBC, anion gap, sodium bicarbonate, BUN, chloride, creatinine, and partial thromboplastin time.

**Figure 5:**
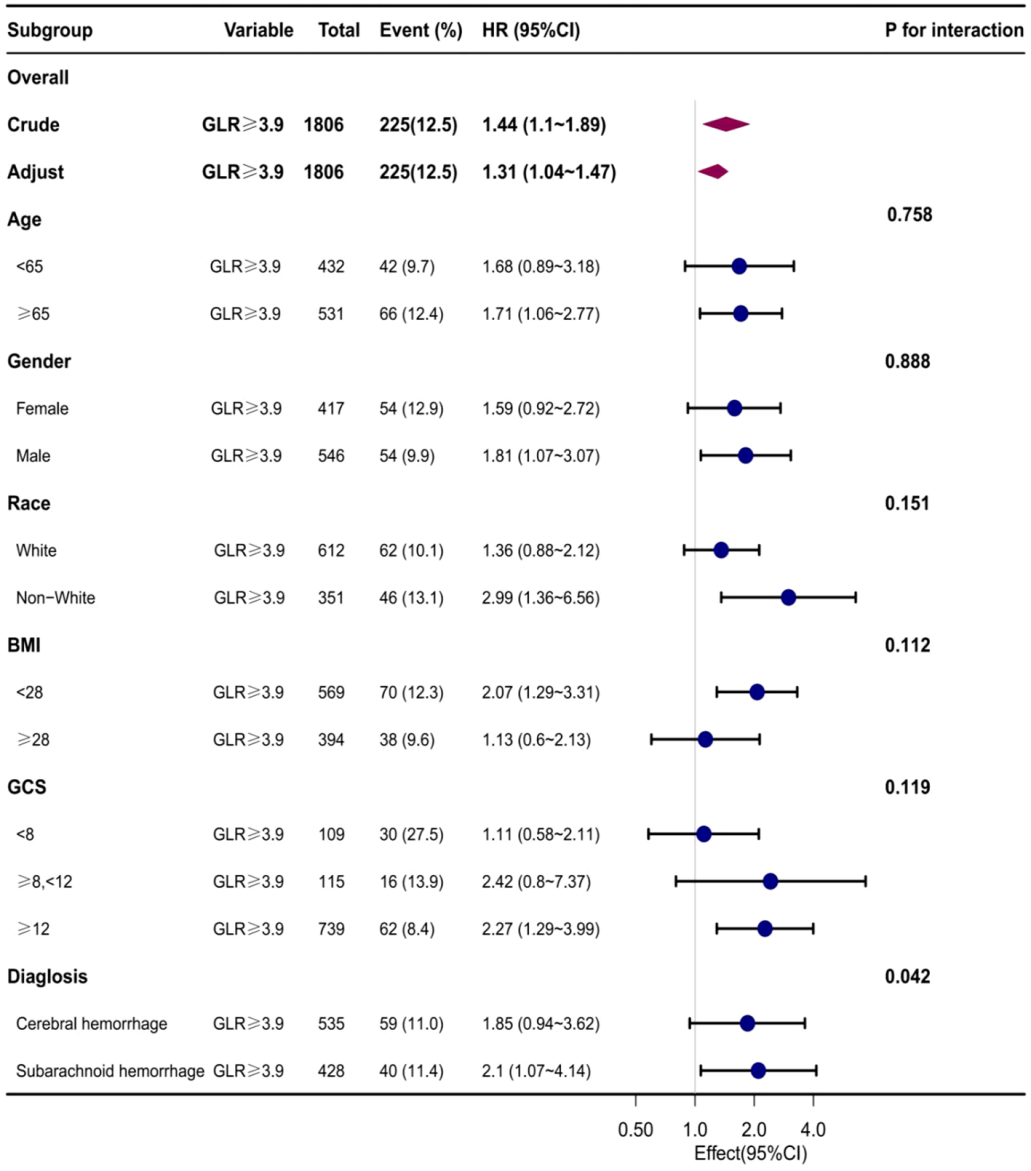
Subgroup analyses of the effect of on Hospital all-cause mortality. Data were adjusted for age, gender, race, BMI, GCS, SOFA, LODS, hemoglobin, Platelets, WBC, anion gap, sodium bicarbonate, BUN, chloride, creatinine, and partial thromboplastin time.

Subgroup analyses were conducted to examine the association between the GLR index and all-cause mortality in both ICUs and hospitals. The findings revealed a statistically significant correlation between elevated GLR index values and increased all-cause mortality rates across various subgroups within ICUs and hospitals, encompassing factors such as age, gender, race, body mass index, Glasgow Coma Scale (GCS) score, and disease type. Specifically, patients classified in the high BAR index group exhibited a significantly heightened risk of all-cause mortality in both ICUs and hospitals when compared to those in the low BAR group, thereby aligning with the overall study outcomes (**Table Supplementary 2**, **3**) (**Figures 4**, **5**).

## Discussion

This study aimed to evaluate the association between ICU mortality and in-hospital mortality among patients with nontraumatic cerebral hemorrhage who were admitted to the intensive care unit, taking into account the GLR and adjusting for variables related to population-based analysis. Our findings showed a significant linear increasing relationship between GLR when used as a continuous variable and ICU mortality and in-hospital mortality among patients with nontraumatic cerebral hemorrhage of all races in the United States. Furthermore, by employing curve fitting techniques, it was determined that the reference point for GLR was 3.9, and the HR trend exhibited consistency on either side of this reference point. When we applied the median to divide them into high and low subgroups and then analyzed them, we found that patients in the high subgroups had higher ICU mortality and in-hospital mortality than those in the low subgroups. And the statistical difference was significant.

Nontraumatic cerebral hemorrhage is distinguished by non-specific metabolic changes in various organs of the central nervous system and the entire body. Prior research has demonstrated that both inflammatory reactions and hyperglycemia play a role in comparable pathophysiological mechanisms subsequent to an incident of cerebral hemorrhage (ICH) (6, 19). Disrupted oxygen consumption, heightened levels of circulating substrates, impaired glucose and lipid oxidation, and malfunctioning mitochondria are linked to multiorgan dysfunction and unfavorable outcomes in animal models and patients. Acute stress can be triggered following a spontaneous cerebral hemorrhage, which disrupts glucose homeostasis and subsequently leads to hyperglycemia (20). This hyperglycemia has detrimental effects on immune function and metabolism, ultimately resulting in adverse outcomes. The mechanisms underlying glucose dysregulation in this context are multifaceted. Furthermore, neuroendocrine stress can lead to hypersecretion of adrenocorticotropic hormone, which affects hyperglycogenism, glucose metabolism, and insulin resistance (21). Additionally, low lymphocyte counts may be correlated with reduced survival time in individuals with nontraumatic cerebral hemorrhage (7).Previously available clinical studies have demonstrated that in individuals with cerebral hemorrhage, the post-onset stimulus is predominantly inflammatory, accompanied by the release of diverse anti-inflammatory cytokines into the bloodstream. This concomitant release of anti-inflammatory cytokines can trigger immunosuppression, resulting in the apoptosis of a substantial number of lymphocytes. Lymphopenia, a prevalent characteristic of immunosuppression following an all-inflammatory response, hinders the clearance of microbes and consequently gives rise to secondary, more severe infections. These infections are the primary cause of mortality among patients with cerebral hemorrhage (6, 12, 22).

The precise mechanism underlying the correlation between increased GLR levels and unfavorable prognosis in individuals with nontraumatic cerebral hemorrhage remains unclear. In recent times, there has been a growing interest among various researchers in integrating blood glucose levels and inflammation-associated lymphocytes to forecast prognostic biomarkers in specific medical conditions (10, 12, 14, 23). Yılmaz A and colleagues (17) have discovered that GLR prior to sorafenib therapy serves as a novel prognostic biomarker, accurately predicting survival rates in patients diagnosed with advanced hepatocellular carcinoma. The prognostic significance of the GLR has been demonstrated in studies involving metastatic gastric cancer (mGC) and metastatic breast cancer (MBC) patients receiving Cdk 4/6 inhibitors (16). Additionally, in patients with type 2 diabetes and MBC, the preoperative hyperglycemia to lymphocyte ratio was found to be an independent predictor of preoperative central lymph node metastasis (18). Constructing a column-line graph could enhance the predictive accuracy of preoperative central lymph node metastasis in these patients. In the realm of research pertaining to cerebral hemorrhage, prior researchers have established a correlation between the neutrophil-to-lymphocyte ratio (NLR) and blood glucose level (BGL), indicating an independent association between the two variables (8, 9). Consequently, it can be inferred that the intricate interplay of various pathological mechanisms potentially influences the progression of inflammatory response and hyperglycemia, thereby exacerbating secondary brain damage. Although the detrimental impact of acute stress and inflammatory response on the outcome of cerebral hemorrhage (ICH) has been acknowledged, the underlying mechanisms remain unidentified (24). The research conducted by Sérgio Fonseca et al. aimed to assess the impact of neutrophil-to-lymphocyte ratio (NLR) on the outcome of intracerebral hemorrhage (ICH), specifically focusing on hematoma expansion and early brain edema (25). Fei Wang et al. analyzed the relationship between neutrophil and lymphocyte ratios and 30-day mortality in patients with acute cerebral hemorrhage, further exploring the role of inflammatory response in disease progression in patients with cerebral hemorrhage (26). While Shaafi S et al. studied the correlation between erythrocyte distribution width, neutrophil-to-lymphocyte ratio, and neutrophil-to-platelet ratio with 3-month prognosis in patients with cerebral hemorrhage, respectively (27, 28). In contrast, our study directly examined the association between the GLR upon admission and mortality rates during hospitalization and in the ICU among nontraumatic cerebral hemorrhage patients admitted to the ICU. We assessed the inflammatory response and glycemic combo of these patients on admission and then conducted a comprehensive analysis of the impact of the main findings.

The potential synergistic effect of septic immune impairment and hyperglycemia should be taken into account when considering the significance of GLR (29, 30). This study presents novel findings on the association between GLR, a readily accessible biomarker, and the mortality rates in ICU and in-hospital settings among patients with nontraumatic cerebral hemorrhage. To the best of our knowledge, this is the first report to establish a distinct correlation between GLR and the mortality rates in both ICU and in-hospital settings among ICU patients with non-traumatic cerebral hemorrhage. This study has the potential to contribute to the development of a diagnostic or predictive model for in-hospital mortality in future research by incorporating the Generalized Linear Regression technique along with other clinical features of spontaneous cerebral hemorrhage. In summary, our study possesses several notable strengths. Firstly, it utilized a large and diverse population to ensure the validity and generalizability of the findings. Second, rigorous statistical adjustments were used in this retrospective observational study to mitigate the effects of potential residual confounding variables. In addition, the implementation of effect-corrected factor analysis improved data utilization and produced more resilient results across subgroups.

There are some noteworthy limitations to this study. Initially, within the MIMIC-IV database, we encountered limitations in acquiring comprehensive data pertaining to calcitoninogen levels, organ function, and the administration of antithrombotic medications among all patients. Consequently, our ability to accurately distinguish between distinct subtypes of cerebral hemorrhage, determine the extent of hemorrhage volumes, and ascertain the usage of antithrombotic medications in the study cohort was compromised. Furthermore, we encountered challenges in obtaining precise information regarding the treatment protocols employed for the enrolled patients, including the administration of ventilation. Additionally, there may exist residual confounding factors that were not accounted for in our analysis. In addition, some patients with nontraumatic cerebral hemorrhage were excluded from our study due to the lack of necessary data, which may have led to biased findings. Secondly, our researchers do not have access to precise treatment protocols and antibiotic use. We contend that this subject holds significant importance and shall serve as the focal point of our forthcoming research endeavors. Thirdly, our study encompassed a population afflicted with severe non-traumatic cerebral hemorrhage, originating solely from a solitary medical facility. Furthermore, it is worth noting that GLR values undergo dynamic fluctuations throughout the course of hospitalization. However, the GLR values utilized in this study were derived from static measurements taken on the initial day of admission to the intensive care unit (ICU) or hospital, rather than from continuously evolving measurements throughout the course of the disease. Consequently, these values solely reflect the impact of the patient’s physical condition at the time of hospital admission on the study outcomes. In light of the retrospective nature of our investigation, which relied on data from the MIMIC-IV database. Therefore, it is imperative to conduct further prospective studies of high quality in order to validate the association between GLR and prognosis. A comprehensive examination of the correlation between prognosis in individuals with nontraumatic cerebral hemorrhage could be undertaken by utilizing sequential BAR measurements as an indicator, when circumstances allow. This endeavor would enhance the prompt detection of severely ill patients within the clinical setting through the utilization of readily accessible biomarkers, thereby facilitating tailored therapeutic interventions aimed at significantly improving patient survival rates.

## Conclusion

In patients with nontraumatic cerebral hemorrhage, GLR were significantly and linearly associated with both ICU mortality and hospital mortality. When GLR was used as a reference point at 3.9, patients in the higher GLR group had significantly higher ICU mortality and hospitalization mortality than those in the lower group.

## Data availability statement

Publicly available datasets were analyzed in this study. This data can be found here: https://mimic.mit.edu/.

## Ethics statement

The studies involving humans were approved by Ethics Committee of Yibin First People’s Hospital, Yibin First People’s Hospital. The studies were conducted in accordance with the local legislation and institutional requirements. The ethics committee/institutional review board waived the requirement of written informed consent for participation from the participants or the participants’ legal guardians/next of kin because This was a retrospective study in a public database, so informed consent was not required.

## Author contributions

SY: Writing – original draft. YL: Data curation, Investigation, Writing – original draft. SW: Data curation, Software, Writing – original draft. ZC: Data curation, Investigation, Writing – original draft. AY: Funding acquisition, Supervision, Writing – review & editing. XH: Supervision, Validation, Visualization, Writing – review & editing.
