# Supplementary material for: Association between high serum blood glucose lymphocyte ratio and all-cause mortality in non-traumatic cerebral hemorrhage: a retrospective analysis of the MIMIC-IV database
Source: Front Endocrinol (Lausanne). 2023 Nov 29;14:1290176. doi: 10.3389/fendo.2023.1290176 (PMC10718300; doi:10.3389/fendo.2023.1290176)
Supplement: Supplementary file 1 [file Table_1.docx]

Table Supplementary1 Univariate analysis of risk factors for ICU mortality and Hospital mortality in patients with nontraumatic cerebral hemorrhage

| **Item** | **ICU all-cause mortality** | | **Hospital all-cause mortality** | |
| --- | --- | --- | --- | --- |
|  | **HR(95%CI)** | ***P-*value** | **HR(95%CI)** | ***P-*value** |
| Age,years | 1.01 (1,1.02) | 0.026 | 1.02 (1.02,1.03) | < 0.001 |
| Gender,Male | 0.72 (0.52,0.99) | 0.045 | 0.84 (0.65,1.09) | 0.2 |
| Race,Non-white | 1.09 (0.78,1.52) | 0.612 | 1.1 (0.84,1.44) | 0.503 |
| BMI,Kg/m^2^ | 0.9997 (0.9718,1.0284) | 0.984 | 0.9947 (0.9719,1.018) | 0.651 |
| Glucose,mg/dl | 1.0015 (1.0007,1.0023) | < 0.001 | 1.0014 (1.0005,1.0023) | 0.002 |
| Lymphocytes | 0.91 (0.78,1.06) | 0.209 | 1.02 (0.91,1.15) | 0.751 |
| SOAI，hours | 1.12 (0.88,1.52) | 0.512 | 1.09(0.94,1.72) | 0.671 |
| GLR | 1.05 (1.02,1.08) | 0.001 | 1.02 (1.01,1.05) | 0.002 |
| GCS | 0.94 (0.91,0.98) | 0.002 | 0.91 (0.88,0.93) | < 0.001 |
| SOFA | 1.17 (1.13,1.21) | < 0.001 | 1.16 (1.13,1.2) | < 0.001 |
| LODS | 1.18 (1.14,1.23) | < 0.001 | 1.2 (1.16,1.24) | < 0.001 |
| SBP,mmHg | 0.98 (0.97,0.99) | < 0.001 | 0.99 (0.98,0.99) | < 0.001 |
| DBP,mmHg | 0.98 (0.96,1) | 0.011 | 0.98 (0.97,0.99) | 0.004 |
| MBP,mmHg | 0.98 (0.96,0.99) | 0.002 | 0.98 (0.97,0.99) | 0.001 |
| RR,beats/min | 1.12 (1.08,1.16) | < 0.001 | 1.01 (1,1.02) | 0.002 |
| HR,beats/min | 1.02 (1.01,1.03) | < 0.001 | 1.09 (1.06,1.12) | < 0.001 |
| Temperature,◦C | 0.76 (0.62,0.92) | 0.006 | 0.77 (0.65,0.92) | 0.004 |
| SpO2, (%) | 0.86 (0.83,0.89) | < 0.001 | 0.86 (0.83,0.9) | < 0.001 |
| Hemoglobin, (g/dL) | 1.01 (0.94,1.09) | 0.734 | 1.04 (0.98,1.1) | 0.204 |
| Platelets, (10^9^/L) | 0.9996 (0.9984,1.0009) | 0.57 | 0.9993 (0.9983,1.0003) | 0.197 |
| WBC, (10^9^/L) | 1.01 (1,1.02) | 0.003 | 1.01 (1.01,1.02) | < 0.001 |
| Anion gap | 1.1 (1.08,1.13) | < 0.001 | 1.08 (1.06,1.1) | < 0.001 |
| Bicarbonate, (mmol/L) | 0.92 (0.88,0.95) | < 0.001 | 0.92 (0.9,0.95) | < 0.001 |
| BUN, (mmol/L) | 1.0094 (1.0044,1.0144) | < 0.001 | 1.0092 (1.005,1.0134) | < 0.001 |
| Chloride, (mmol/L) | 1.004 (0.9799,1.0286) | 0.748 | 1.0052 (0.9852,1.0257) | 0.61 |
| Creatinine, (mmol/L) | 1.08 (1.01,1.15) | 0.034 | 1.04 (0.98,1.1) | 0.184 |
| Sodium, (mmol/L) | 1.03 (1,1.06) | 0.051 | 1.03 (1,1.06) | 0.027 |
| PTT, seconds | 1.0034 (0.9992,1.0076) | 0.115 | 1.0021 (0.9984,1.0058) | 0.26 |

SOAI，stroke onset to the admission to ICU;GLR:serum blood glucose/lymphocyte count; BMI,Body mass index;SBP, systolic blood pressure; DBP, diastolic blood pressure; MBP, mean blood pressure; RR, respiratory rate; HR, heart rate; SpO2, percutaneous oxygen saturation; GCS, Glasgow Coma Score;SOFA,Sequential organ function score;LODS,The Logistic organ dysfunction system;WBC, white blood cell; BUN, blood urea nitrogen;PTT,Plasma prothrombin time.
