# Supplementary material for: Association between high serum blood glucose lymphocyte ratio and all-cause mortality in non-traumatic cerebral hemorrhage: a retrospective analysis of the MIMIC-IV database
Source: Front Endocrinol (Lausanne). 2023 Nov 29;14:1290176. doi: 10.3389/fendo.2023.1290176 (PMC10718300; doi:10.3389/fendo.2023.1290176)
Supplement: Supplementary file 3 [file Table_3.docx]

Table Supplementary3 Subgroup analysis of high GLR and in-hospital mortality in patients with nontraumatic cerebral hemorrhage

| **Subgroup** | **Variable** | **n.total** | **n.event(%)** | **Followup.**  **Time** | **HR(95CI%)** | ***P*-value** | ***P* for interaction** |
| --- | --- | --- | --- | --- | --- | --- | --- |
| Age<65 |  |  |  |  |  |  | 0.758 |
|  | GLR<3.9 | 392 | 15 (3.8) | 1269.89 | 1(Ref) |  |  |
|  | GLR≥3.9 | 432 | 42 (9.7) | 1526.89 | 1.68 (0.89~3.18) | 0.111 |  |
| Age≥65 |  |  |  |  |  |  |  |
|  | GLR<3.9 | 451 | 25 (5.5) | 1696.03 | 1(Ref) |  |  |
|  | GLR≥3.9 | 531 | 66 (12.4) | 2015.12 | 1.71 (1.06~2.77) | 0.028 |  |
| Gender(Female) |  |  |  |  |  |  | 0.888 |
|  | GLR<3.9 | 346 | 19 (5.5) | 1158.68 | 1(Ref) |  |  |
|  | GLR≥3.9 | 417 | 54 (12.9) | 1519.98 | 1.59 (0.92~2.72) | 0.094 |  |
| Gender(Male) |  |  |  |  |  |  |  |
|  | GLR<3.9 | 497 | 21 (4.2) | 1807.24 | 1(Ref) |  |  |
|  | GLR≥3.9 | 546 | 54 (9.9) | 2022.03 | 1.81 (1.07~3.07) | 0.026 |  |
| Race(White) |  |  |  |  |  |  | 0.151 |
|  | GLR<3.9 | 588 | 32 (5.4) | 2049.54 | 1(Ref) |  |  |
|  | GLR≥3.9 | 612 | 62 (10.1) | 2237.26 | 1.36 (0.88~2.12) | 0.166 |  |
| Race(Non-White) |  |  |  |  |  |  |  |
|  | GLR<3.9 | 255 | 8 (3.1) | 916.38 | 1(Ref) |  |  |
|  | GLR≥3.9 | 351 | 46 (13.1) | 1304.75 | 2.99 (1.36~6.56) | 0.006 |  |
| BMI<28 |  |  |  |  |  |  | 0.112 |
|  | GLR<3.9 | 531 | 25 (4.7) | 1959.32 | 1(Ref) |  |  |
|  | GLR≥3.9 | 569 | 70 (12.3) | 2069.58 | 2.07 (1.29~3.31) | 0.002 |  |
| BMI≥28 |  |  |  |  |  |  |  |
|  | GLR<3.9 | 312 | 15 (4.8) | 1006.6 | 1(Ref) |  |  |
|  | GLR≥3.9 | 394 | 38 (9.6) | 1472.43 | 1.13 (0.6~2.13) | 0.694 |  |
| GCS<8 |  |  |  |  |  |  | 0.119 |
|  | GLR<3.9 | 90 | 18 (20) | 517.9 | 1(Ref) |  |  |
|  | GLR≥3.9 | 109 | 30 (27.5) | 718.24 | 1.11 (0.58~2.11) | 0.758 |  |
| GCS<12,≥8 |  |  |  |  |  |  |  |
|  | GLR<3.9 | 98 | 5 (5.1) | 619.77 | 1(Ref) |  |  |
|  | GLR≥3.9 | 115 | 16 (13.9) | 581.32 | 2.42 (0.8~7.37) | 0.119 |  |
| GCS≥12 |  |  |  |  |  |  |  |
|  | GLR<3.9 | 655 | 17 (2.6) | 1828.25 | 1(Ref) |  |  |
|  | GLR≥3.9 | 739 | 62 (8.4) | 2242.45 | 2.27 (1.29~3.99) | 0.005 |  |
| Cerebral hemorrhage |  |  |  |  |  |  | 0.042 |
|  | GLR<3.9 | 468 | 41 (8.8) | 1159.38 | 1(Ref) |  |  |
|  | GLR≥3.9 | 535 | 77 (14.4) | 1338.09 | 1.85 (0.94~3.62) | 0.074 |  |
| Subarachnoid hemorrhage |  |  |  |  |  |  |  |
|  | GLR<3.9 | 375 | 43 (11.5) | 1001.25 | 1(Ref) |  |  |
|  | GLR≥3.9 | 428 | 64 (15.0) | 1231.19 | 2.1 (1.07~4.14) | 0.032 |  |
